# Honeybee Venom Immunotherapy: A Comparative Study Using Purified and Nonpurified Aqueous Extracts in Patients with Normal Basal Serum Tryptase Concentrations

**DOI:** 10.1155/2012/869243

**Published:** 2012-01-12

**Authors:** M. Beatrice Bilò, Barbara Cinti, M. Feliciana Brianzoni, M. Chiara Braschi, Martina Bonifazi, Leonardo Antonicelli

**Affiliations:** Allergy Unit, Department of Allergy, Immunology and Respiratory Diseases, University Hospital Ospedali Riuniti, Via Conca 1, 60020 Ancona, Italy

## Abstract

In this study, we compared a purified aqueous extract and the corresponding nonpurified aqueous preparation under the same build-up protocol in bee venom allergic patients with a normal baseline mast cell tryptase concentration. Eighty patients with a history of a systemic reaction were enrolled for immunotherapy using a 5-day rush protocol. Patients treated with the purified extract and those treated with the non purified aqueous extract who developed a systemic reaction underwent maintenance therapy with the purified aluminium hydroxide adsorbed preparations. Patients treated with the nonpurified aqueous extract who did not experience a systemic reaction during the rush phase underwent the maintenance phase with that extract. Systemic reactions during the build-up phase occurred significantly more often in patients treated with nonpurified aqueous extract than in those treated with the corresponding purified aqueous preparations. During the one-year maintenance phase, no systemic reactions occurred in either of the groups. Neither age nor baseline mast cell tryptase concentration presented a significant correlation with the occurrence of a systemic reaction during the treatment, while the type of extract did. In conclusion, nonpurified aqueous extracts induced more frequent systemic reactions than the purified aqueous preparations, during the same rush protocol. The efficacy seemed to be comparable.

## 1. Introduction

Subcutaneous VIT with a standard dose of 100 *μ*g is highly effective treatment [1, 2]. The indications for VIT are a history of an SR, positive venom skin or serum-specific IgE antibodies [1, 3], the knowledge of the natural history and risk factors for severe outcome [4], and impaired quality of life [5]. VIT can however have side effects, the most serious being anaphylaxis [1, 6]. HB venom and the build-up phase are well-known risk factors for side effects during VIT. Although there are no definite conclusions regarding the role of the dose increase schedule [6, 7], in a recent European multicentre study of 680 VIT patients, the rapid dose increase (ultrarush > rush > conventional phase) appears to be a risk factor for side effects during the build-up phase [8]. Moreover, mast cell diseases and also an elevated bSTC represent other risk factors [8, 9]. In Europe, VIT may be performed using PA and NPA venom extracts and PAHA preparations (depot preparations). The latter of the three is used in the conventional build-up and maintenance phases, while the aqueous preparations are used in ultra-rush, rush, clustered, and maintenance phases. Many European specialists switch from the aqueous extracts to PAHA preparations after updosing, whether or not they cause side effects [10]. The efficacy of the PA and PAHA extracts is supported by studies using both sting challenge and in-field stings and is comparable to that of nonpurified preparations [11]. In comparative trials, both PA and PAHA extracts appear to be better tolerated than NPA extracts, especially in the prevention of severe large local reactions (LLRs) [11, 12].

As PA extracts and the corresponding NPA extracts in HBV-allergic patients have not been compared so far, this study aims to prospectively compare treatment safety in terms of SRs by administering PA and the corresponding NPA bee-venom extracts during the same rush phase in patients with a normal bSTC and evaluate the safety of depot versus NPA extracts during a one-year maintenance phase.

## 2. Material and Methods

### 2.1. Patients

Consecutive HBV-allergic patients with a history of a SR ≥ Mueller grade II [13] were enrolled for VIT with a PA or the corresponding NPA extract. A diagnosis of HBV allergy was made if the patient had a history of an SR to a honeybee sting, was positive to skin testing, and/or had serum specific IgE to *Apis mellifera* venom [3]. Pregnancy and beta-blocker treatment were considered as standard exclusion criteria [3], as well as mastcell diseases and also a bSTC ≥ 11.5 *μ*g/L (Immunofluorimetric CAP assay, Unicap 100; Phadia, Uppsala, Sweden).

The Local Ethics Committee approved the Study Protocol and patients gave their written informed consent.

### 2.2. Allergen Extracts for VIT

The following extracts were used for treatment: (1) the traditional NPA HBV preparations (Pharmalgen ALK-Abellò aqueous extracts, HØrsholm, Denmark), reconstituted in albumin-containing saline diluent; (2) a PA HBV preparation (Aquagen SQ, ALK-Abellò); (3) a purified aluminium-adsorbed HBV depot preparation (Alutard SQ, ALK-Abellò). All preparations contain 100 *μ*g/mL of allergens. PA venom extracts are products with no low-molecular components present in the native venom extract (cutoff: 1000 D). Purification is mainly obtained through a Sephadex-gel filtration process, which allows separation of the protein fractions by means of their molecular weight. PA venom extracts do not contain vasoactive amines like dopamine, histamine, and serotonin. In addition, a gel-filtration procedure reduces the presence of small peptides like apamin, kinins, and mast cell degranulating peptides in the final product. In the PAHA extract, the raw venom underwent the same purification procedure with recovery of the allergen-containing fraction only and subsequently adsorbed onto aluminium hydroxide.

### 2.3. Treatment Regimens

The patients underwent a 5-day rush VIT regimen (Table 1) with the aqueous preparations and were split into groups A (40 patients) and B (40 patients), which were administered NPA (Pharmalgen ALK-Abellò) and PA extracts (Aquagen SQ, ALK-Abellò), respectively. Patients experiencing SRs during the build-up phase from both groups were switched from the aqueous to the PAHA extract and received weekly interval injections of up to 100 *μ*g of venom. During the maintenance phase, group A patients who developed no rush-phase SRs went on to receive maintenance therapy with the non-purified aqueous extract while group B patients who tolerated the build-up phase were also treated with the PAHA preparation. Maintenance VIT was performed in both groups at 4 weekly intervals for one year. The patients were not administered premedication.

### 2.4. Adverse Reactions

Only those patients with a history of anaphylaxis undergoing rush VIT were hospitalised in our clinic. The other patients were kept on an outpatient regimen on the day of treatment. Injections were administered in our clinic by the same allergist. SRs to VIT injections were Mueller classified [13].

Development of an SR during the rush-phase necessitated suspending treatment until the patient made a complete recovery, after which a depot extract was used. In the event of a large local or a mild SR, the VIT dosage was maintained. Weekly interval injections up to the standard maintenance dosage were then administered.

Patients were instructed to immediately report any suspected delayed VIT-related reactions experienced at home.

### 2.5. Efficacy

The use of hospital sting challenges remains an ethical issue in Italy and is generally avoided for testing VIT efficacy. This being the case, patients were instructed to report details of any reaction to a field sting and any pharmacological treatment. To assess treatment efficacy in non beekeepers, only those stings typically attributable to bees, recognisable by the embedded stinger at the sting site, were considered.

### 2.6. Statistics and Sample Size Calculation

Statistical analysis was carried out using SPSS statistical software (vers. 13) (SPSS Chicago, IL, USA). Differences between the two groups in outcome variables (i.e., occurrence of SRs) were assessed using Fisher's exact test (whenever an expected cell value was <5) and the *χ*-square test. Relative risks with 95% CI were also calculated. The number needed to harm (NNH) was calculated according to McQuay and Moore [14]. A multiple-logistic regression analysis was performed to identify clinical and demographic variables (age, gender, reaction severity, baseline STC, type of VIT, purified versus non-purified) associated with the occurrence of SRs during VIT. The unpaired *t*-test and Mann-Whitney test, when appropriate, were used to compare quantitative variables. A two-sided *P* value < 0.05 was considered significant for all analyses.

The power calculation assumed a difference between PA and NPA treated subjects in the incidence of SRs of at least 65% less (relative risk reduction). This assumption provided 80% power at an alpha level of 0.05 for a sample size of at least 40 evaluable patients per group. Sample size calculation was performed using GPower Statistical Software ver 3.03 (Germany).

## 3. Results

### 3.1. Patients

**Figure 1:**
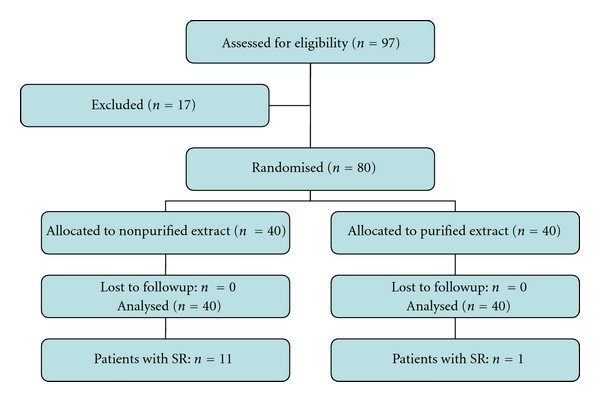
Study flow for the build-up phase of VIT.

**Figure 2:**
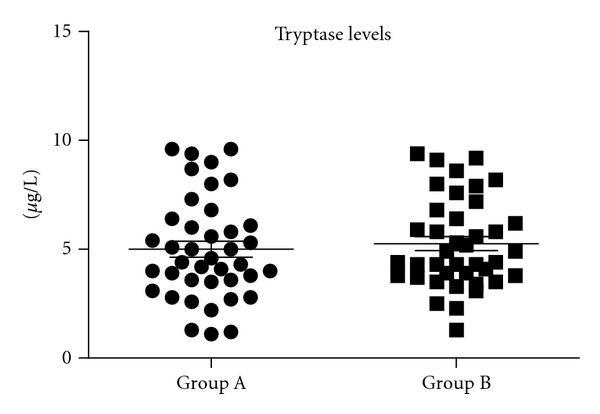
Baseline serum tryptase level (*μ*g/L) in patients treated with purified aqueous HB venom extract (Group A) and in patients treated with non-purified aqueous preparation (Group B).

Ninety-seven consecutive patients with a history of an SR to HB stings were evaluated for the study, of whom 17 (17.5%) were excluded on the grounds of a bSTC ≥ 11.5 *μ*g/L (Figure 1), leaving 80 to be allocated to two treatment groups: 40 in group A and 40 in group B. Prior to VIT, 16 patients (20%) had experienced a grade II SR, 28 patients (35%) a grade III SR, and 36 patients (45%) a grade IV SR. The two groups of patients (cases and controls) were comparable with respect to age, sex, bSTC and SR severity (Table 2 and Figure 2).

### 3.2. Adverse Reactions

#### 3.2.1. Dose Increase Phase

**Figure 3:**
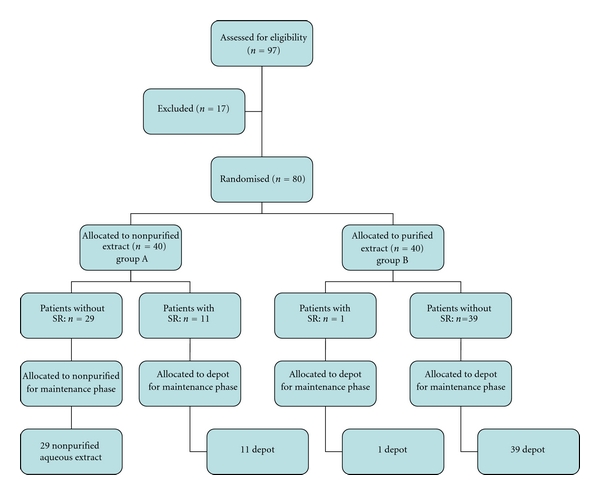
Study flow for the build-up and maintenance phases of VIT.

The cumulative doses during the tolerated 5-day rush-phase was 223.11 *μ*g of HBV. Eleven (27.5%) (95%CI: from 13 to 41%) group A patients developed an immediate SR: 6 were grade I reactions and 5 grade II (Table 3). Treatment was suspended in these patients until they fully recovered, and in almost without exception VIT was resumed with the equivalent dosage of the PAHA extract. A maintenance dose of 100 *μ*g was reached by weekly interval injections, with no side effects. Thirty-nine group B patients tolerated the rush-phase and then switched to the PAHA extract for the HBV 100 *μ*g maintenance therapy with no side effects (Figure 3). One (2.5%) (95% CI: from 2 to 7%) group B patients developed a grade I SR (Table 3) and switched from the aqueous purified extract to the PAHA extract (Figure 3), with no SR, successfully reaching the maintenance dose of 100 *μ*g, with the result that patients treated with the NPA extract during the rush phase experienced significant more frequent SRs than those who underwent VIT with the PA extract (*P* = 0.0017 Fisher's exact test). The absolute risk reduction was 25%, and the relative risk (purified versus nonpurified extracts) was 91% (95% CI from 100 to 53) with a NNH of 4.2. In the rush induction phase, the incidence of SRs per total number of injections (i.e 634) was 0.1%/doses in the purified-extract-treated patients (group A) compared to 1.9%/doses in the nonpurified-extract-treated group of patients (total number of injections 555) (*P* = 0.0017 Chi-squared test). No patient in group A or B experienced late SRs.

#### 3.2.2. Maintenance Phase

Maintenance dose was 100 *μ*g in all patients. Twenty-nine group A patients underwent one-year maintenance treatment with the NPA extract, while 51 (11 group A and 40 group B patients) were given the PAHA extract (Figure 3). No SRs occurred.

### 3.3. Tryptase Dosage

**Figure 4:**
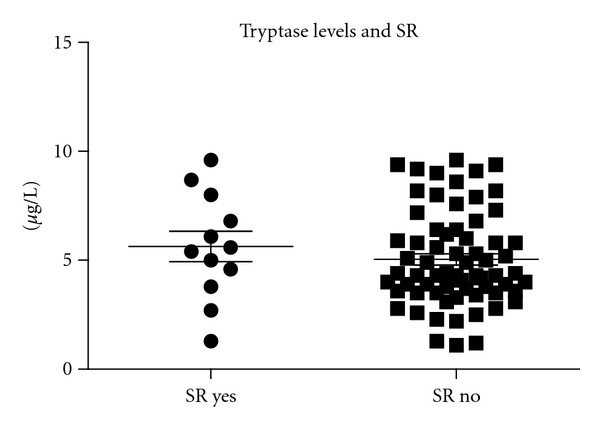
Baseline serum tryptase level in patients who developed a systemic reaction (SR) during the build-up phase of VIT in comparison with those who did not.

The mean (SD) value of bSTC was 5 (±2.3) *μ*g/L (median 4.5) and 5.3 (±2) *μ*g/L (median 4.7) in group A and B patients, respectively (*P* = n.s.) (Figure 2).The mean value of bSTC was 5,6 *μ*g/L (median 5.5) in patients who developed SRs and 5.3 *μ*g/L (median 5.4) in those who did not (*P* = n.s.) (Figure 4). Multiple logistic regression analysis did not reveal a significant correlation between the occurrence of an SR during VIT with age, gender, reaction severity, or basal STCs, while, in contrast, the type of extract (NPA) correlated significantly (*P* = 0.0001).

### 3.4. Efficacy

No patient was re-stung during the build-up phase. Thirteen out of the 29 (44.8%) NPA extract treated patients were re-stung during the one year maintenance phase without developing a reaction.

Fifteen out of the 51 patients (13.6%) treated with the PAHA extract during the maintenance phase were re-stung without side effects, of whom one developed a mild SR during VIT.

## 4. Discussion

While probably being the most effective form of allergen immunotherapy currently available to physicians [1, 2, 10], subcutaneous VIT is at the same time able to improve health-related quality of life [5]. However, the treatment can cause side effects ranging from an LLR to a severe SR [1]. A recent paper comparing purified preparations with non-purified extracts reviewed the literature on the respective safety and effectiveness of the two [12]. The authors found that in comparative trials purified extracts appear to be better tolerated than non-purified extracts, while PAHA extracts seem to be safer than the corresponding PA preparation, especially in the prevention of severe LLRs. Also, they concluded that further prospective-controlled studies are needed in order to evaluate the ability of purified extracts to reduce the frequency of SRs over the corresponding non-purified preparation [12].

This is the first study comparing the safety of purified and the corresponding non-purified aqueous venom preparations in terms of SRs in HBV-allergic patients with a normal bSTC under the same build-up protocol. We studied HV-venom-allergic patients as their risk of developing an SR during VIT is greater than that of vespid-allergic patients [1]. The scope of our paper is confined to the study of VIT-induced SRs. LLRs, though frequent and bothersome, do not usually necessitate a dose reduction and do not prevent from reaching the full maintenance dose. They are also no risk factor for SRs to later injections.

Patients with a bSTC ≥ 11.5 *μ*g/L were excluded as this factor itself is a potential risk for side effects during VIT. Indeed, a recent paper reports that a rise in the bSTC from 4.21 *μ*g/L to 20 *μ*g/L only in vespid allergic patients is accompanied by a simultaneous increase in requirement for emergency intervention during the build-up phase by a factor of approximately 3.75 [8].

In this study, we compared the same build-up protocol in two homogeneous groups of patients who were treated with two different extracts from the same company. We have demonstrated that patients treated with the non-purified HBV experienced significantly more SRs than those treated with the purified HBV preparation, due to a higher occurrence of SRs during the build-up phase. In fact, during the same 5-day rush phase, 27.5% of the NPA-extracts-treated patients developed an SR compared with 2.5% of the PA-extract-treated subjects (*P* = 0.0017; NNH = 4.2). Reactions were nonsevere with six patients developing a grade I and five a grade II reaction. The lower frequency of SRs following injections of the PA preparations compared with the NPA extracts, as demonstrated by our study, may be due to the former's purification of low-molecular-weight irritants, with recovery of the allergen-containing fraction only. This finding cannot be attributed to the cumulative dose applied over a given span of time as the two groups of patients underwent the same 5-day rush protocol.

Patients who had SRs during the build-up phase were switched to the PAHA extract from the NPA solution, as were those treated with the PA extract whether they had experienced an SR or not. During the one-year maintenance phase, no SRs occurred in either group. Though there was no control group, our data indirectly confirm the safer profile of the PAHA extracts. In fact, all those patients who experienced PA- or NPA-induced SRs during the rush phase could have benefited from being switched to the same dosage of a PAHA preparation and have subsequently achieved the 100 *μ*g maintenance dose with weekly interval injections.

To date, the only study which compares PA and PAHA extracts with the corresponding NPA preparations dates back to 1986 and was done in yellow-jacket-venom- (YJV-) allergic patients. The authors demonstrated that PA-induced SRs were milder than those caused by NPA preparations, but more frequent with the PAHA extracts [15]. In HBV-allergic patients, a few studies compare the PAHA extract with the PA extract under different protocols [16–18], but none with the corresponding NPA extract. In HBV-allergic patients, Rueff demonstrated that treatment with the PA extract caused significantly more frequent LLRs than the depot extract during the 5-day rush and maintenance phases. On the other hand, though not significant, aqueous preparations induced SRs more frequently during the updosing phase [16].

Another study of HBV-allergic patients, in which the safety of the PA and PAHA extracts and of three different induction protocols were compared, demonstrated that depot cluster VIT was better tolerated than PA rush VIT, which in turn was less well tolerated than the PA cluster protocol [18].

The efficacy of the PA and PAHA preparations is supported by studies implementing sting challenges in HBV- and YJV-allergic patients and is comparable to that of non-purified extracts [12]. In-hospital sting challenges are the only reliable means of evaluating the effect of VIT [1]. This procedure, however, remains an ethical issue in Italy, making in-field stings the only available feedback on the efficacy of the treatment. Though the observational period was over one year and only a few patients had in-field restings, our experience shows that the efficacy of the non-purified and purified extracts is highly comparable.

Our study suffers from a few limitations which need to be taken into account from a results point of view. Firstly, we conducted an unblinded open trial. Despite this, the main outcome was the incidence of SRs which should be considered as the “hard” end point. Also, the clinical definition of SR is well defined and accepted with a low margin to subjectivity. Secondly, the sample size required to verify the trial hypothesis was calculated in advance and the relative risk reduction (RRR) in the number of SRs we observed (−91%) in the purified extract-treated patients was far greater than the RRR forecast in calculation of the sample size (−65%).

In conclusion, our study demonstrates the superiority of the PA extract in HBV-allergic subjects over the corresponding non-purified extracts under the same rush incremental phase.

Though similar in terms of their efficacy, HB PA extracts are safer option than NPA preparations for specialists who perform rush VIT, followed by maintenance treatment with the PAHA preparation. The use of the HB depot extracts for the conventional incremental and maintenance phases could be proposed as a workable solution for specialists with less experience in managing VIT.

Moreover, in patients with SRs caused by both PA and NPA extracts, the switch to a PAHA extract could be safely made and allow the generally adequate maintenance dose of 100 *μ*g of venom to be reached.

## Figures and Tables

**Table 1:** Protocol of VIT build-up rush phase.

Day	Dose	mL administered	*μ*g administered	Conc *μ*g/mL
1	1	0.10	0,01	0,1
	2	0.10	0,1	1
	3	0.10	1	10
	4	0.20	2	10

2	5	0.30	3	10
	6	0.35	3,5	10
	7	0.35	3,5	10

3	8	0.10	10	100
	9	0.15	15	100
	10	0.15	15	100

4	11	0.20	20	100
	12	0.25	25	100
	13	0.25	25	100

5	14	0.30	30	100
	15	0.35	35	100
	16	0.35	35	100

**Table 2:** Demographic and clinical data of patients included in the study.

	Group A (Nonpurified) *N* = 40	Group B (purified) *N* = 40	*P* values
Sex (male/female)	34/6	30/10	0.8

Age, years, mean (SD)	42 (17)	42 (16)	0.8

Mueller classification (Reaction to HB sting)			
Grade I	0	0	0.7
Grade II	7	9	
Grade III	15	13	
Grade IV	18	18	

**Table 3:** Systemic reaction during the build-up phase of VIT.

Build-up 5-day rush phase			
Group	No. of patient	VIT day	SR*
(A) Aqueous non-purified	2	2	II
	1	2	I
	3	3	II
	3	3	I
	1	4	I
	1	5	I

(B) Aqueous purified	1	3	I

*According to Mueller classification [13].

## References

[1] Bonifazi F, Jutel M, Biló BM, Birnbaum J, Muller U (2005). Prevention and treatment of hymenoptera venom allergy: guidelines for clinical practice. *Allergy*.

[2] Golden DBK, Moffitt J, Nicklas RA (2011). Stinging insect hypersensitivity: a practice parameter update 2011. *Journal of Allergy and Clinical Immunology*.

[3] Biló BM, Rueff F, Mosbech H (2005). Diagnosis of hymenoptera venom allergy. *Allergy*.

[4] Bilò MB, Bonifazi F (2009). The natural history and epidemiology of insect venom allergy: clinical implications. *Clinical and Experimental Allergy*.

[5] Oude Elberink JNG, De Monchy JGR, Van Der Heide S, Guyatt GH, Dubois AEJ (2002). Venom immunotherapy improves health-related quality of life in patients allergic to yellow jacket venom. *Journal of Allergy and Clinical Immunology*.

[6] Mosbech H, Muller U (2000). Side-effects of insect venom immunotherapy: results from an EAACI multicenter study. *Allergy*.

[7] Birnbaum J, Ramadour M, Magnan A, Vervloet D (2003). Hymenoptera ultra-rush venom immunotherapy (210 min): a safety study and risk factors. *Clinical and Experimental Allergy*.

[8] Ruëff F, Przybilla B, Biló MB (2010). Predictors of side effects during the buildup phase of venom immunotherapy for hymenoptera venom allergy: the importance of baseline serum tryptase. *Journal of Allergy and Clinical Immunology*.

[9] Niedoszytko M, De Monchy J, Van Doormaal JJ, Jassem E, Oude Elberink JNG (2009). Mastocytosis and insect venom allergy: diagnosis, safety and efficacy of venom immunotherapy. *Allergy*.

[10] Bilò BM, Bonifazi F (2011). Hymenoptera venom immunotherapy. *Immunotherapy*.

[11] Bilò MB, Severino M, Cilia M (2009). The VISYT trial: venom immunotherapy safety and tolerability with purified versus nonpurified extracts. *Annals of Allergy, Asthma and Immunology*.

[12] Bilò MB, Antonicelli L, Bonifazi F (2010). Purified versus nonpurified venom immunotherapy. *Current Opinion in Allergy and Clinical Immunology*.

[13] Mueller HL (1966). Diagnosis and treatment of insect sensitivity. *The Journal of Asthma Research*.

[14] McQuay HJ, Moore RA (1997). Using numerical results from systematic reviews in clinical practice. *Annals of Internal Medicine*.

[15] Mosbech H, Malling H-J, Biering I (1986). Immunotherapy with yellow jacket venom. A comparative study including three different extracts, one adsorbed to aluminium hydroxide and two unmodified. *Allergy*.

[16] Ruëff F, Wolf H, Schnitker J, Ring J, Przybilla B (2004). Specific immunotherapy in honeybee venom allergy: a comparative study using aqueous and aluminium hydroxide adsorbed preparations. *Allergy*.

[17] Alessandrini AE, Berra D, Rizzini FL (2006). Flexible approaches in the design of subcutaneous immunotherapy protocols for hymenoptera venom allergy. *Annals of Allergy, Asthma and Immunology*.

[18] Quercia O, Emiliani F, Pecora S, Burastero SE, Stefanini GF (2006). Efficacy, safety, and modulation of immunologic markers by immunotherapy with honeybee venom: comparison of standardized quality depot versus aqueous extract. *Allergy and Asthma Proceedings*.

